# Pump–probe capabilities at the SPB/SFX instrument of the European XFEL

**DOI:** 10.1107/S1600577522006701

**Published:** 2022-07-21

**Authors:** Jayanath C. P. Koliyadu, Romain Letrun, Henry J. Kirkwood, Jia Liu, Man Jiang, Moritz Emons, Richard Bean, Valerio Bellucci, Johan Bielecki, Sarlota Birnsteinova, Raphael de Wijn, Thomas Dietze, Juncheng E, Jan Grünert, Daniel Kane, Chan Kim, Yoonhee Kim, Max Lederer, Bradley Manning, Grant Mills, Luis L. Morillo, Nadja Reimers, Dimitrios Rompotis, Adam Round, Marcin Sikorski, Cedric M. S. Takem, Patrik Vagovič, Sandhya Venkatesan, Jinxiong Wang, Ulrike Wegner, Adrian P. Mancuso, Tokushi Sato

**Affiliations:** a European XFEL, Holzkoppel 4, 22869 Schenefeld, Germany; b School of Chemical and Physical Sciences, Keele University, Staffordshire ST5 5AZ, United Kingdom; c Center for Free Electron Laser Science, Deutsches Elektronen-Synchrotron, Notkestraße 85, 22607 Hamburg, Germany; d Department of Chemistry and Physics, La Trobe Institute for Molecular Science, La Trobe University, Melbourne, Victoria 3086, Australia; RIKEN SPring-8 Center, Japan

**Keywords:** pump–probe experiments, European XFEL, megahertz pump and probe sources, time-resolved experiments

## Abstract

Pump–probe experiments at X-ray free-electron laser (XFEL) facilities are a powerful tool for studying dynamics at ultrafast and longer timescales. Observing the dynamics in diverse scientific cases requires optical laser systems with a wide range of wavelength, flexible pulse sequences and different pulse durations, especially in the pump source. Here, the pump–probe instrumentation available for measurements at the Single Particles, Clusters, and Biomolecules and Serial Femtosecond Crystallography (SPB/SFX) instrument of the European XFEL is reported. The temporal and spatial stability of this instrumentation is also presented.

## Abstract 2

The pump–probe capabilities at the SPB/SFX instrument of the European XFEL are discussed.

## 1. Introduction

The advent of hard X-ray free-electron lasers (XFELs) with high peak intensities and femtosecond pulse durations has paved a way to study the interaction between matter and light at ultrafast time scales and to atomic resolution (Emma *et al.*, 2010 ▸; Ishikawa *et al.*, 2012 ▸; Kang *et al.*, 2017 ▸; Decking *et al.*, 2020 ▸; Prat *et al.*, 2020 ▸). Ultrafast dynamics that are triggered and observed using an X-ray pulse or a secondary source of radiation, in general known as pump–probe (PP) experiments, are of increasing utility and have become a general method at XFEL facilities worldwide (Fukuzawa & Ueda, 2020 ▸; Jang *et al.*, 2020 ▸; Pandey *et al.*, 2020 ▸; Biasin *et al.*, 2021 ▸). In most cases, the dynamics of interest are triggered or ‘pumped’ using an optical laser and observed or ‘probed’ by an XFEL (Zhang *et al.*, 2014 ▸; Kim *et al.*, 2015 ▸; Lemke *et al.*, 2017 ▸; Jung *et al.*, 2021 ▸. More recently, the development of superconductive accelerator technology has allowed some XFELs to operate at a high repetition rate (Feldhaus, 2010 ▸; Rossbach, 2020 ▸; Decking *et al.*, 2020 ▸) and this offers the potential to reduce the data acquisition time by several orders of magnitude compared with non-superconducting free-electron lasers (Gisriel *et al.*, 2019 ▸; Sobolev *et al.*, 2020 ▸; Ayyer *et al.*, 2021 ▸). The European XFEL (EuXFEL) is the only presently operating high-repetition-rate XFEL in the hard X-ray regime. However, EuXFEL operates in the so-called ‘burst mode’ with a burst duration of 600 µs at a repetition rate of 10 Hz. Each burst can have a single pulse to a train of pulses with an intra-burst repetition rate up to 4.5 MHz. This demands more advanced technology to stabilize variations in beam properties, largely caused by non-thermal equilibrium of the mechanical components involved in the generation, transport and conditioning of the pump and the probe sources.

Here we report the pump–probe capabilities and parameters available to study a wide variety of scientific cases at the Single Particles, Clusters, and Biomolecules and Serial Femtosecond Crystallography (SPB/SFX) instrument of the European XFEL.

## 2. Pump–probe experiments at SPB/SFX

The SPB/SFX instrument is primarily focused on imaging as well as determining the structure of single particles and macro-molecules using hard X-rays (Mancuso *et al.*, 2019 ▸). Various experiments based on coherent diffraction imaging (Sobolev *et al.*, 2020 ▸; Ayyer *et al.*, 2021 ▸), serial femtosecond X-ray crystallography (Wiedorn *et al.*, 2018 ▸; Grünbein *et al.*, 2018 ▸; Yefanov *et al.*, 2019 ▸) and X-ray imaging (Vagovič *et al.*, 2019 ▸) have been performed to successfully visualize structures across a variety of length scales, utilizing megahertz repetition rate X-ray pulses. Exploring the ultrafast dynamics of biochemical reactions and time-resolved behavior of molecular structures are also being studied at SPB/SFX (Pandey *et al.*, 2020 ▸). To facilitate such experiments at the instrument, different optical laser pump sources and diagnostic devices have been installed and commissioned.

**Figure 1:**
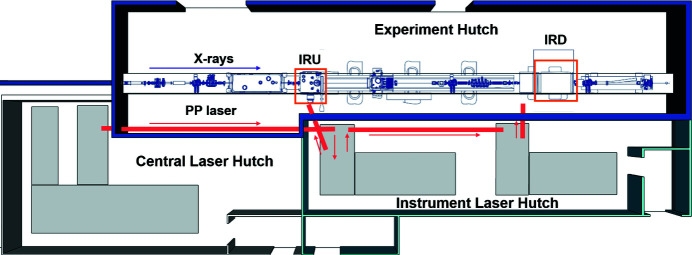
Top view of the general layout of the SPB/SFX instrument showing the central laser hutch, instrument laser hutch and experiment hutch. The two interactions regions are highlighted by orange boxes, the PP laser beam pipes and beam directions are marked in red, and the X-ray beam, marked in violet, goes from left to right.

The SPB/SFX instrument has two interaction regions where sample interactions with X-rays and laser sources are measured, one termed interaction region upstream (IRU) and the other termed interaction region downstream (IRD) (Mancuso *et al.*, 2019 ▸). The general layout of the SPB/SFX instrument is shown in Fig. 1 ▸. The X-rays propagate from the source to their focal point at IRU entirely through a vacuum, arriving with a full width at half-maximum (FWHM) focused diameter of a few micrometres or a few hundred nanometres using either of two Kirkpatrick–Baez mirror systems (Bean *et al.*, 2016 ▸). A set of compound refractive lens refocusing optics (providing a micrometre-sized focus) are installed after IRU to enable measurements at IRD in an air/helium or vacuum environment (Mancuso *et al.*, 2019 ▸). Pump–probe experiments are feasible at both interaction regions. A MHz X-ray imaging setup is also partially installed and commissioned, including a fast camera [Shimadzu HPV-X2 (Shimadzu, 2021 ▸)] and an X-ray imaging unit around IRD (Vagovič *et al.*, 2019 ▸); further developments are ongoing. This paper primarily describes the capability currently available at IRU with a similar capability planned for IRD in the future.

**Figure 2:**
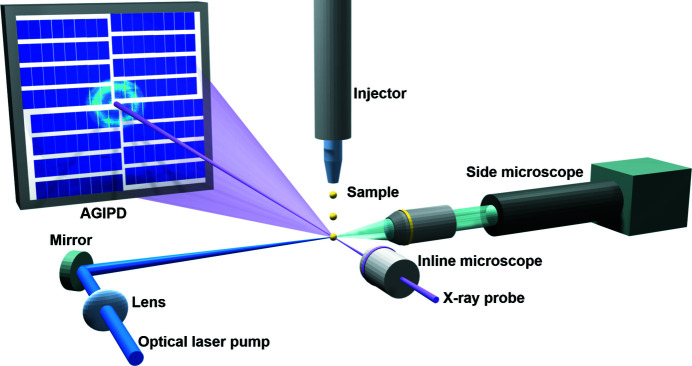
Schematic of a pump–probe experimental setup at IRU of the SPB/SFX instrument.

Fig. 2 ▸ shows a simplified drawing of a typical in-vacuum pump–probe experiment at IRU. The samples are delivered to the interaction region via a liquid jet or an aerosol injector (Schulz *et al.*, 2019*a* ▸; Bielecki *et al.*, 2019 ▸; Vakili *et al.*, 2022 ▸). The SPB/SFX instrument geometry is suited for off-axis excitation with a laser perpendicular to the X-ray beam. The focus and pointing of the laser beam can be adjusted using a focusing lens and a steering mirror using piezo stages. The typical optical laser beam size at the interaction point is 30–50 µm FWHM. Optimization of the laser and X-ray focus, and their spatial overlap, are monitored with microscopes which provide orthogonal views. The diffraction pattern is recorded with the 1 megapixel adaptive gain integrating pixel detector (AGIPD), a fast, burst-mode compatible X-ray detector (Allahgholi *et al.*, 2015 ▸).

The main diagnostics devices at IRU are side and inline microscopes (Mancuso *et al.*, 2019 ▸). The inline microscope consists of an infinity corrected 10× reflective objective (Edmund Optics, ReflX Objective, 89-724), a prism mirror, a tube lens (Thorlabs, AC254-200-AB-ML) and a CMOS camera (Basler ace acA2500-14gm). The secondary mirror of the reflective objective and the prism mirror have a 700 µm and 2 mm hole, respectively, to let the X-ray beam through. The side microscope consists of an objective or lens combined with a tube lens (Thorlabs, TTL200) and an sCMOS camera (Andor, Zyla 5.5) in air. Up to three objectives/lenses can be mounted on a motorized stage to provide different magnifications of the interaction point. The most commonly used optics is 10× (10× Mitutoyo Plan Apo Infinity Corrected Objective, 378-803-3). The inline and side microscopes were designed to provide a working distance of >30 mm to accommodate the requirements of the sample environment (Schulz *et al.*, 2019*a* ▸).

## 3. Femtosecond PP laser system

**Figure 3:**
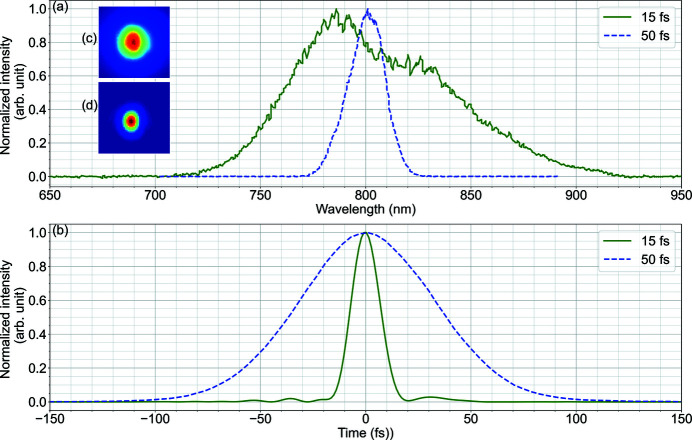
Spectral (*a*) and temporal (*b*) profile of the PP laser at 15 fs FWHM and 50 fs FWHM pulse duration. The insets show the beam profile in the near-field (*c*) and far-field (*d*).

The principal pump–probe laser (PP laser) at SPB/SFX is a burst-mode laser developed by the laser group at the European XFEL (Pergament *et al.*, 2016 ▸; Palmer *et al.*, 2019 ▸), delivering ultrashort pulse duration with up to 4.5 MHz intra-burst repetition rate. The PP laser is based on a non-collinear optical parametric amplification (NOPA) system which is able to provide various choices of pulse duration and wavelength tunability around 800 nm. The PP laser system consists of a femtosecond oscillator, a burst-mode fibre-based front-end and a power amplifier followed by up to three NOPA stages. The different NOPA stages can be set up according to the required output parameters at the instrument (Palmer *et al.*, 2019 ▸). The PP laser can be operated with the same pulse pattern as the XFEL; however, there is also a means to select only a subset of pulses within the burst. Therefore, any arbitrary pulse pattern derived from the intra-burst repetition rate can be generated. In addition, it is possible to use a sequence of varying pulse patterns for consecutive bursts – that is, different patterns for subsequent trains. The NOPA pump beam can also be used as an independent source, which gives options of uncompressed 400 ps and compressed 850 fs pulses at 1030 nm (Palmer *et al.*, 2019 ▸). The output parameters of the PP laser are tabulated in Table 1 ▸. The spectral and temporal profiles of the compressed 800 nm pulses for the 15 fs FWHM and 50 fs FWHM pulse duration set points are shown in Fig. 3 ▸.

The PP laser is transported from the central laser hutch (CLH) to the SPB/SFX instrument laser hutch (ILH) through a 10.4 m-long pipe under vacuum. Relay imaging is used to maintain pointing stability over the long propagation distance. Both temperature (21 ± 0.1°C) and humidity (50 ± 2.5%) in the ILH are regulated to minimize the impact of environmental fluctuations. Additionally, the PP laser beam path and optics are kept in a laser-safe enclosure in order to reduce the effect of fluctuations in airflow in both the ILH and experiment hutch. The laser beam size, pulse energy and optical delay are conditioned in the ILH before transport to IRU through a 1.4 m-long vacuum pipe without relay imaging optics. The total optical path length from the output of the NOPA to IRU is about 40 m. When the PP laser is operated at the 800 nm/15 fs setpoint, pulses are initially negatively chirped to a duration of about 300 fs and compressed to the desired pulse duration close to the interaction point using fused silica glass plates with anti-reflection coating at 800 nm. For the 800 nm/50 fs setpoint, the pulse duration is optimized at the desired location by means of a Treacy compressor (Treacy, 1969 ▸). Currently, the PP laser is only available at IRU in SPB/SFX, and in general is limited to single-wavelength operation during a given experiment.

**Figure 4:**
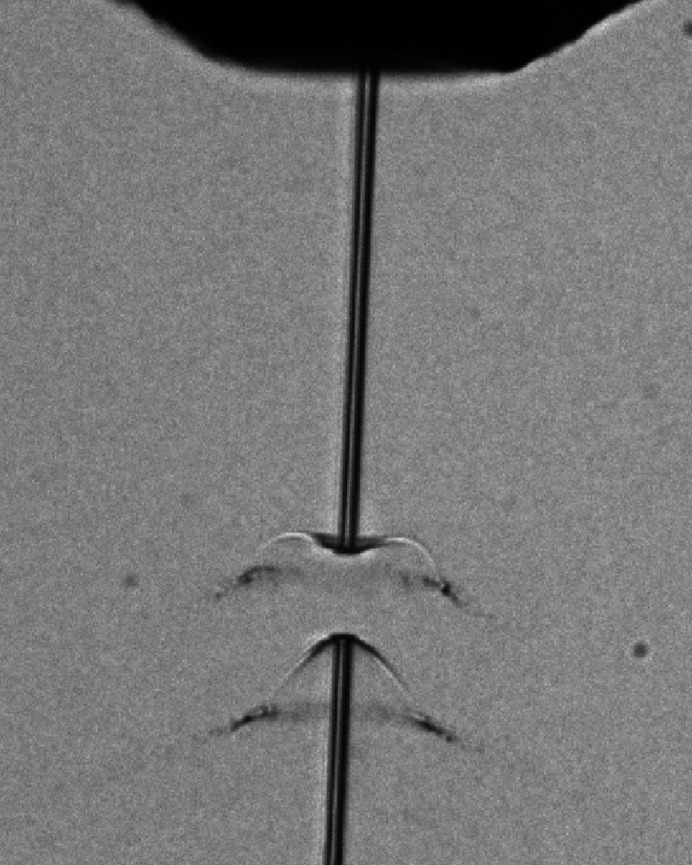
Visualizing the water jet explosion induced by an intense X-ray pulse with the PP laser. The image was captured by the side microscope with a 20× objective (20× Mitutoyo Plan Apo SL Infinity Corrected Objective, 378-810-3).

The 800 nm branch of the PP laser is split into two in the ILH with 99% transported to the interaction region and the remaining 1% used for diagnostics. This enables online monitoring of not only beam profile, pointing stability, pulse-resolved intensity and spectrum but also the arrival time relative to X-ray pulses via a photon arrival time monitor (Kirkwood *et al.*, 2019 ▸; Sato *et al.*, 2020 ▸; Letrun *et al.*, 2020 ▸). The PP laser may also be used for ‘stroboscopic image’ samples at the interaction region (Kay & Wheeless Jr, 1976 ▸; Schropp *et al.*, 2015 ▸; Reich *et al.*, 2018 ▸). Stroboscopic imaging is used to optimize liquid jet alignment with respect to the X-rays and to determine the velocity of the liquid jet *in situ* (Vakili *et al.*, 2022 ▸), and also enables imaging the dynamics of liquid jets/droplets exposed to intense X-ray pulses (Gorel *et al.*, 2020 ▸). An example of PP laser stroboscopic imaging to capture the water jet explosion induced by a X-ray pulse is shown in Fig. 4 ▸.

## 4. Pulse structure/pattern of optical lasers

**Figure 5:**
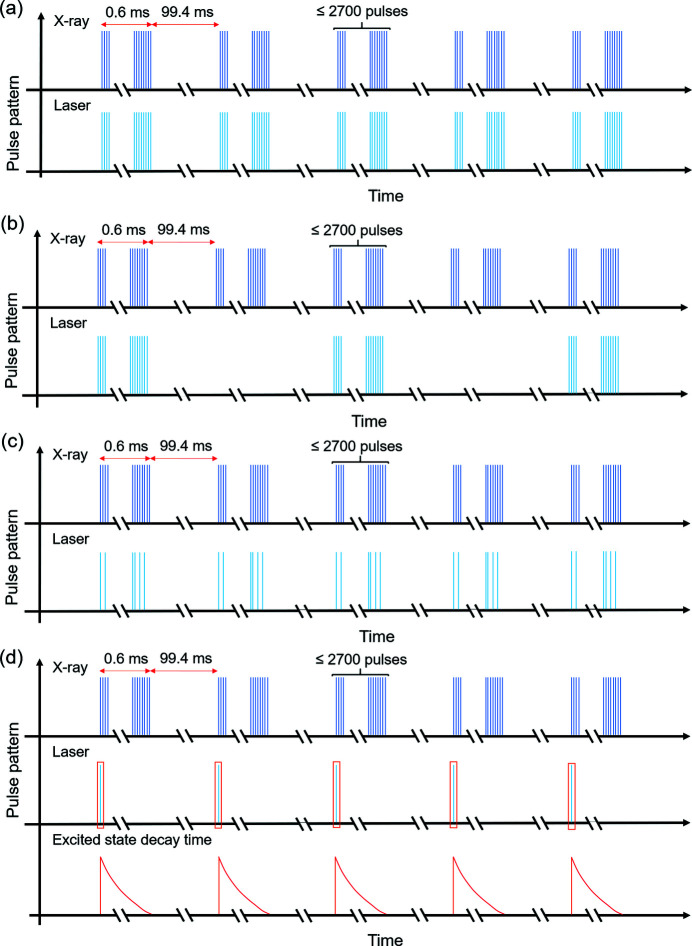
Several examples of pulse pattern setting for optical laser pulses. (*a*) Pump and probe source have the same pulse pattern. (*b*) Burst-mode operation where the optical laser pulses are down picked for so-called light and dark states for consecutive trains. (*c*) Burst-mode excitation with an arbitrary intra-train pulse pattern derived from a megahertz X-ray pulse pattern. (*d*) 10 Hz pump and sampling with a megahertz X-ray pulse pattern.

In a typical pump–probe experiment at SPB/SFX, dynamics can be studied at a wide range of timescales using a variety of pulse sequences. Pulse sequences are arranged by controlling the pulse pattern of the PP laser with respect to the X-rays. For the 800 nm output from the parametric amplifier of the PP laser, the pulse picking is realized with an acousto-optic modulator (AOM) installed before supercontinuum generation (Palmer *et al.*, 2019 ▸). For the 1030 nm output of the PP laser, the pulse pattern is controlled by a BBO Pockels cell placed behind the booster power amplifier of the pump beam for the parametric amplifier (Palmer *et al.*, 2019 ▸). The burst length of the NOPA pump beam is usually adjusted to cover the length of the applied laser pattern. No obvious pointing drift or beam profile changes were observed with different pump burst lengths. Fig. 5 ▸(*a*) shows a schematic of a typical operation mode where the optical laser pulse train has the same structure as the XFEL pulse train. Here a burst-mode excitation with both megahertz optical laser excitation and megahertz X-ray probe is illustrated. This optical laser pulse structure within the burst can be tuned from single shot (10 Hz) to 4.5 MHz. This operation mode offers a precise control over the temporal delay between optical laser and X-ray pulses and this delay can be extended up to the pulse period, *i.e.* 220 ns in the case of a 4.5 MHz repetition rate. In burst-mode excitation, the optical laser pulses can also be picked for certain trains as shown in Fig. 5 ▸(*b*) instead of for all the trains as shown in Fig. 5 ▸(*a*). The example shows picked alternate trains of optical laser pulses, with a megahertz burst mode for both the optical laser and X-ray pulses, resulting in X-ray pulse trains at 10 Hz and optical laser pulse trains at 5 Hz. The pulse picking capability of the optical laser allows user-defined arbitrary pulse patterns derived from the intra-burst repetition rate of the optical laser. Fig. 5 ▸(*c*) shows an arbitrary pulse pattern for the optical laser and periodic pulse pattern for X-rays at a megahertz intra-burst repetition rate.

Using the commercial nanosecond lasers (discussed in Section 7), samples can be pumped at 10 Hz with single- or multi-stage pump schemes, suitable for tracking relatively slow dynamics up to sub-millisecond timescales [shown in Fig. 5 ▸(*d*)]. In a more limited set of cases the pulse picking capability of the PP laser can also be used for multi-stage pump schemes. A corresponding control of the X-ray pulse pattern is also possible (Obier *et al.*, 2019 ▸).

## 5. Wavelength extension with optical parametric amplifier

**Figure 6:**
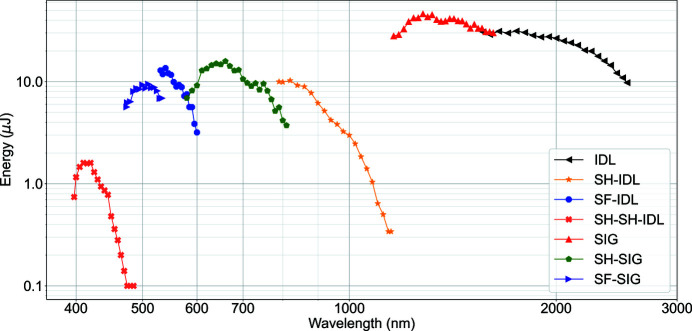
Output of TOPAS at different wavelengths. For this measurement, TOPAS was pumped by the PP laser with the following parameters: 800 nm, ∼54 fs, 215 µJ and 50 pulses per train at 1.1 MHz. (SH – second harmonic; SF – sum frequency; SIG – signal; IDL – idler).

A commercial optical parametric amplifier (OPA) was recently commissioned at SPB/SFX. The OPA is a TOPAS Prime (Traveling-Wave Optical Parametric Amplifier of White-Light Continuum) from Light Conversion (LightConversion, 2017 ▸) with additional modules, providing the possibility to tune the wavelength from 400 nm to 2600 nm. The TOPAS is located in the ILH and can be pumped using the PP laser with the following parameters: 800 nm, 50–60 fs and an intra-burst repetition rate of 100 kHz to 1.1 MHz. The pulse energy measured at the output of the TOPAS over the possible deliverable tuning range is shown in Fig. 6 ▸.

## 6. Stability of the PP laser system

**Figure 7:**
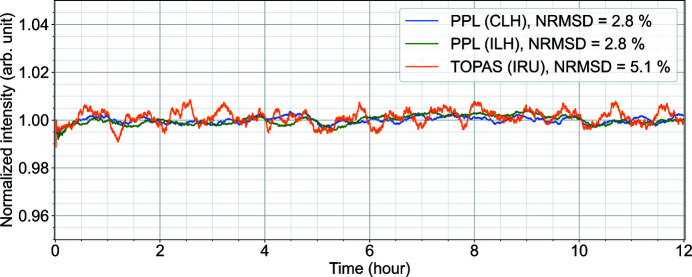
Intensity stability of the TOPAS output measured at IRU over 12 h with the output beam set at a wavelength of 640 nm. The three lines show the 10 min moving average of intensity stability of the PP laser in the CLH, the ILH and the OPA output at IRU. Here the intensity is normalized to the mean intensity of each measurement.

The intensity and pointing stability of the PP laser and OPA output are an important factor in the execution of successful pump–probe experiments combined with micrometre to sub-micrometre focused X-rays. The PP laser is routed and conditioned by a large number of optics from the CLH to IRU for a total path length of about 40 m. Transport over such a long distance can easily introduce fluctuations in laser beam pointing. Hence it is essential to investigate positional and intensity stability at the interaction point. The TOPAS output beam, 640 nm at 1.1 MHz intra-burst repetition rate, was focused to a smaller than 50 µm FWHM diameter spot using a plano-convex lens (*f* = 300 mm) at IRU. The pointing of this beam was measured at the sample position in IRU by the side microscope at an acquisition rate of 10 Hz over a period of 12 h. The microscope image data were processed and fitted with a 1D Gaussian, along both horizontal and vertical directions, to estimate the beam diameter and central position. Each acquired image from the microscope was integrated to obtain the intensity stability data for the TOPAS beam at IRU. The intensity stability of the PP laser fundamental was also measured in the CLH and the ILH over the same period by imaging the beam using a CCD. The intensity stability measurements in ILH and CLH were based on the maximum pixel intensity in the CCD for each image acquired. Fig. 7 ▸ shows the intensity stability of the PP laser fundamental and the OPA. The normalized root-mean-square deviation (NRMSD) of the TOPAS output intensity over 12 h at IRU was 5.1%.

**Figure 8:**
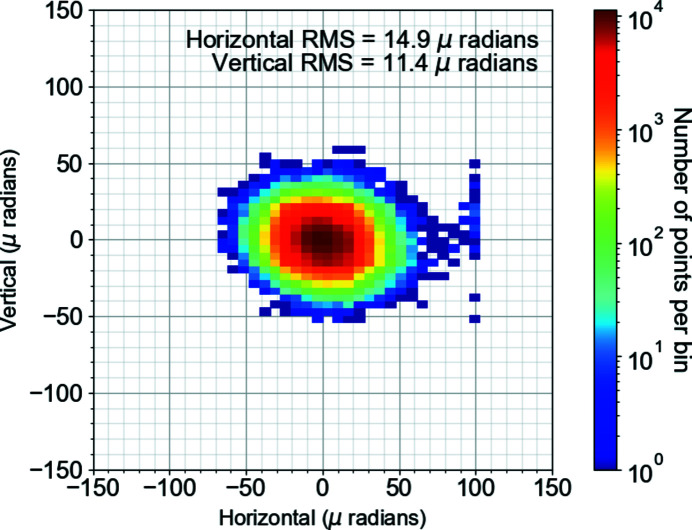
2D histogram of the pointing stability of the TOPAS output beam in the horizontal and vertical directions.

Fig. 8 ▸ shows the pointing stability of the TOPAS measured over 12 h as a 2D histogram. The beam pointing is estimated from the beam center, obtained from processing the image, divided by the focal length of the lens used to focus the beam to the interaction region. The beam pointing jitters were 14.9 µrad r.m.s. and 11.4 µrad r.m.s. in the horizontal and vertical directions, respectively. This corresponds to 5.4% and 5% of the mean beam diameter, measured as 49 µm and 40 µm, in the horizontal and vertical directions, respectively. Due to this positional stability, no active beam stabilization setup was installed for the PP laser.

## 7. Nanosecond laser systems

In addition to the PP laser, with its femtoscond to picosecond pulse duration, it is also possible to pump or excite samples with laser pulses of longer pulse duration using nanosecond laser systems at SPB/SFX. There are two types available, either fixed wavelength or continuously tunable.

The tunable nanosecond lasers are commercial systems based on an optical parametric oscillator (OPO), Opollette HE 355 LD (OPOTEK), with a tuning range of 210 nm to 2200 nm. Three identical laser systems are installed and each has a repetition rate of up to 20 Hz, a pulse duration of 7–9 ns, and their beams are delivered via optical fiber to the interaction regions. These three lasers are synchronized with the 10 Hz trigger derived from the EuXFEL master clock (Lamb *et al.*, 2019 ▸) and can be used simultaneously to excite/illuminate the samples with a desired pulse pattern. The maximum pulse energy for these laser systems is of the order of a few mJ.

The fixed-wavelength nanosecond lasers are commercial Nd:YAG lasers from Litron Lasers, Nano LG 150-10 and Nano LG 300-10, that generate pulses with maximum energies of 150 mJ and 300 mJ, respectively, at 10 Hz. The fundamental wavelength for these two lasers is 1064 nm, and their second and third harmonics are also available. These relatively high power lasers can be placed close to the interaction regions and the laser beam can be transported to the interaction region in free space.

**Figure 9:**
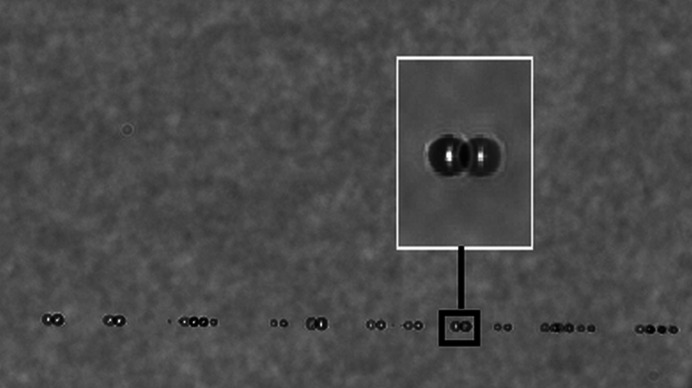
Double exposure stroboscopic imaging of droplets with two nanosecond lasers with a delay of hundreds of nanoseconds between the two pulses. A zoomed view of one of the droplets imaged is shown in the inset. The image was collected using the side microscope with a 10× objective and an Andor Zyla 5.5 camera.

**Figure 10:**
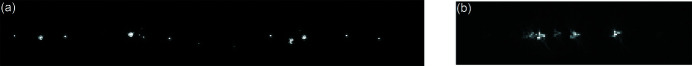
An aerosol particle beam with particles of size of a few tens of nanometres visualized by Rayleigh scattering imaging using the (*a*) inline microscope with 10× objective and (*b*) side microscope with 2× objective.

The pulses from either type of nanosecond laser can be delivered to both IRU and IRD. These nanosecond lasers, like the PP laser, may also be used as a light source for stroboscopic imaging of samples (Kay & Wheeless Jr, 1976 ▸; Schropp *et al.*, 2015 ▸; Reich *et al.*, 2018 ▸; Vakili *et al.*, 2022 ▸), as shown in Fig. 9 ▸. The high-power nanosecond lasers can be used to visualize particles which have a size of hundreds of nanometres or smaller in focused aerosol beams, by detection of the Rayleigh scattering due to the particles (Hantke *et al.*, 2018 ▸). This imaging technique is routinely used for the alignment of an aerosol sample’s particle beam to the tightly focused X-ray beam. An example for aerosol particle beam imaging using Rayleigh scattering is shown in Fig. 10 ▸.

## 8. Synchronization and photon arrival time monitor

The EuXFEL uses an all-optical synchronization system to achieve high timing accuracy of all sub-systems throughout the facility (Lamb *et al.*, 2019 ▸; Schulz *et al.*, 2019*b* ▸). A radio-frequency (RF) synchronization system, with lower timing fidelity, is also provided for initial timing synchronization (Kirkwood *et al.*, 2019 ▸). The EuXFEL facility uses a main RF oscillator with a frequency of 1.3 GHz, synchronized to the main laser oscillator, which provides the reference optical signals for the entire optical synchronization system. The main laser oscillator is a passively mode-locked commercial laser with a wavelength of 1550 nm, pulse duration of 200 fs and repetition rate of 216.7 MHz (Schulz *et al.*, 2019*b* ▸). The timing signals from the main laser oscillator are transported via polarization-maintaining optical fibers, with the fiber length stabilized by temperature control. The PP laser is synchronized using a subsidiary laser oscillator that is synchronized to the main laser oscillator and is located in the timing station near the instruments. The typical temporal stability of the PP laser with respect to the all-optical synchronization system is ≤5 fs r.m.s. when optically synchronized (Palmer *et al.*, 2019 ▸).

**Figure 11:**
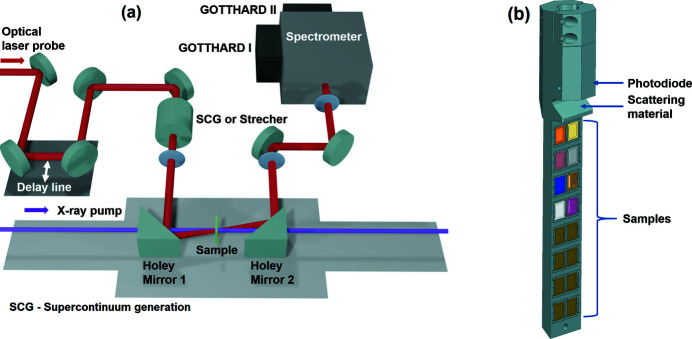
Schematic of (*a*) the PAM setup, and (*b*) the PAM sample holder.

The photon arrival time monitor (PAM) prototype was developed and installed in collaboration with the EuXFEL X-ray photon diagnostics group (Liu *et al.*, 2017 ▸). It employs a spectral encoding method (Bionta *et al.*, 2011 ▸, 2014 ▸) to measure the relative time delay between PP laser and X-rays (Harmand *et al.*, 2013 ▸; Hartmann *et al.*, 2014 ▸). Fig. 11 ▸(*a*) shows a schematic of the PAM setup at SPB/SFX. The PAM is located between the two interaction regions of the SPB/SFX instrument. The PP laser optical path length of the IRU and PAM branches are set such that the timing is coincident with the X-ray pulses at both IRU and PAM. Although the 800 nm, 15 fs PP laser may be used directly in the PAM, in practice a supercontinuum is generated in a sapphire plate to increase the spectral bandwidth and for a quasi-flat-top spectral profile. The PAM sample holder is capable of mounting up to 16 materials with various thicknesses. This enables the choice of the optimal material for timing measurement for a given X-ray photon energy and fluence. The presence of spares also ensures that the material can be quickly exchanged if it is damaged by either the laser or X-ray beams. In general, the spatial overlap between X-ray and laser pulses is optimized with a sample that exhibits intense luminescence upon X-ray irradiation, such as Ce:YAG. A coarse timing overlap and monitoring is achieved with a fast photodiode (G4176-03, HAMAMATSU) which is installed on top of the PAM sample holder in such a way as to monitor elastically scattered X-rays from a material on the PAM sample holder. A schematic of the sample holder is shown in Fig. 11 ▸(*b*). For sub-picosecond timing overlap and monitoring, the X-ray beam pumps a thin dielectric material, *e.g.* Ce:YAG or Si_3_N_4_, and the X-ray induced change of the optical properties of the material is then probed by the supercontinuum laser pulses. The laser pulses transmitted through the sample are coupled into a commercial spectrometer (Andor, Shamrock 193i) with dual output ports. Each port is equipped with a line GOTTHARD detector (Mozzanica *et al.*, 2012 ▸) that is capable of recording up to 800 kHz in its burst-mode setting (Mozzanica *et al.*, 2012 ▸; Zhang *et al.*, 2014 ▸). Here, the GOTTHARD detectors are set up at 564 kHz each with interleaved acquisition windows to allow acquisition at a repetition rate of up to 1.13 MHz (Sato *et al.*, 2020 ▸; Letrun *et al.*, 2020 ▸).

**Figure 12:**
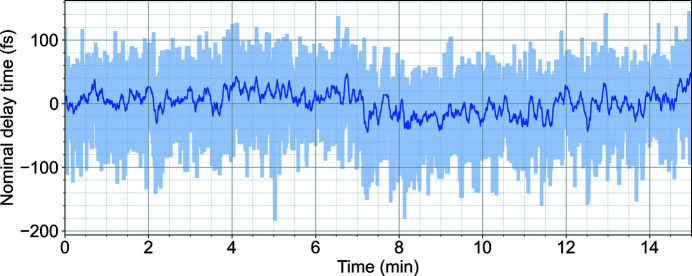
Plot of temporal jitter measured over 15 min. The darker blue line shows the rolling mean over 5 s (Sato *et al.*, 2020 ▸).

The temporal jitter between X-ray and laser pulses with optical synchronization was measured at a 1.13 MHz repetition rate at the SPB/SFX instrument using the PAM. The r.m.s. jitter from train to train, for a given pulse, was measured to be <25 fs over a period of 10 min (Sato *et al.*, 2020 ▸; Letrun *et al.*, 2020 ▸). Fig. 12 ▸ shows a plot of temporal jitter between X-ray and laser pulses measured over 15 min.

## 9. Alignment procedure for pump–probe experiments at SPB/SFX

The general alignment procedure for pump–probe experiments at SPB/SFX requires optimization of both the spatial and temporal overlap of the X-rays and laser pulses.

Typically, a Ce:YAG scintillator of thickness 20 µm is used to visualize the X-ray position and ensure the spatial overlap of the laser and X-rays. The experimental geometry used is similar to the pump–probe geometry shown in Fig. 2 ▸ with the liquid sample jet replaced by a Ce:YAG scintillator.

The X-ray beam size and position are usually visualized and measured using the inline microscope with the Ce:YAG scintillator oriented normal to the incident X-ray beam. For the determination of the spatial and temporal overlap of X-ray and laser pulses, the scintillator is rotated to increase the angle of incidence of the X-ray beam to 70°–75°, with the two surfaces of the scintillator then facing the incident laser beam and side microscope optics, respectively.

**Figure 13:**
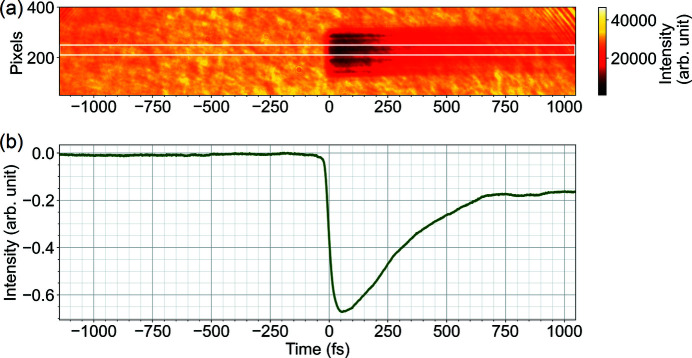
Time zero position determination using spatial encoding at SPB/SFX. (*a*) Laser transmission imaged with the side microscope with 10× magnification. (*b*) Line-out of the selected region highlighted by the white box in (*a*).

A coarse temporal overlap, or so-called ‘time zero’, between X-ray and laser pulses on the picosecond timescale is achieved using a fast photodiode. A finer temporal overlap between X-ray and laser beams at the interaction region is determined using a method called spatial encoding (Harmand *et al.*, 2013 ▸). The time point where the delay between laser and X-ray pulses is zero or falls within the detection limit, defined as ‘time zero’ in this manuscript, is optimized using a mechanical optical delay line in the laser beam path. The time zero position can be determined by a decrease in transmission of the laser pulse through a material induced by the X-ray pulse excitation, and imaged by the side microscope. Fig. 13 ▸ shows a typical image taken by the side microscope to determine time zero at the interaction region. This method relies on the same phenomenon as the spectral encoding used in the PAM; however, with the timing information encoded in the spatial rather than in the spectral domain (Maltezopoulos *et al.*, 2008 ▸; Harmand *et al.*, 2013 ▸).

The PAM can then be aligned to the time zero at the interaction region and be used for measuring and monitoring the temporal jitter and drift during a pump–probe experiment.

After the optimization of spatial and temporal overlap of X-ray and laser pulses, the sample of interest is aligned to the optimized spatial overlap position at the interaction point within a few micrometres. These samples are then excited with optical laser pulses and probed with X-ray pulses with the required time delay between the two. Jitter between the optical and X-ray pulses can be measured and recorded with the PAM on a shot-to-shot basis.

## 10. Conclusion

The pump–probe capabilities and general alignment procedure for a pump–probe experiment at the SPB/SFX instrument of the European XFEL are presented here. The available optical laser parameters are tabulated in Table 1 ▸.

The temporal jitter at the instrument between X-rays and PP laser is of the order of a few tens of femtoseconds, and the femtosecond output of the PP laser at IRU has around 5% fluctuation in both intensity and pointing over 12 h. The SPB/SFX instrument provides high-stability optical pump sources with a variety of wavelengths with up to megahertz repetition rates. At SPB/SFX, the temporal delay between X-ray and laser can be measured and monitored with the PAM. With both high precision of timing as well as a high data rate, the SPB/SFX instrument is a versatile tool to study ultrafast phenomena. The unique pulse structure of both the X-ray and laser sources at the European XFEL, and the possibility to change these pulse patterns independently, makes the instrument an ideal place to study longer timescale dynamics in addition to the ultrafast timescales.

## Figures and Tables

**Table 1:** Available optical laser parameters at SPB/SFX For the PP laser, the repetition rate is the intra-burst repetition rate; the pulse energy depends on this repetition rate setting. SH – second harmonic. TH – third harmonic. FH – fourth harmonic.

Laser type	Wavelength	Pulse duration	Pulse energy	Repetition rate
PP laser	800 nm (750–850 nm) + SH and TH	15, 50 or 300 fs	0.05–2.5 mJ	10 Hz to 4.5 MHz
PP laser	1030 nm + SH, TH and FH	0.85 or 400 ps	1–40 mJ	10 Hz to 4.5 MHz
TOPAS (OPA)	400–2600 nm	50–100 fs	Up to 20 µJ	10 Hz to 1.1 MHz
ns laser (OPO)	210–2200 nm	7–9 ns	Up to 5 mJ	Up to 20 Hz
ns laser (Nd:YAG)	1064 nm + SH and TH	7–9 ns	Up to 300 mJ	10 Hz
